# The Epidemiology of Sport-Related Spinal Cord Injuries in the Gulf Region: A Systematic Review

**DOI:** 10.7759/cureus.62141

**Published:** 2024-06-11

**Authors:** Amin G Gronfula, Ali Alhaddad, Thamer H Alsharif, Raef F Alamri, Yaser Alhassan, Lamees Alghdali, Ahmad A Kalantn, Fahad Abduljabbar

**Affiliations:** 1 Orthopedic Surgery, Al-Noor Hospital, Makkah, SAU; 2 Orthopedic Surgery, East Jeddah General Hospital, Jeddah, SAU; 3 Neurosurgery, Royal College of Surgeons in Ireland, Dublin, IRL; 4 Orthopedics and Traumatology, Al-Noor Hospital, Makkah, SAU; 5 Orthopedics, Royal College of Surgeons in Ireland, Dublin, IRL; 6 Medicine, Royal College of Surgeons in Ireland, Dublin, IRL; 7 Orthopedic Surgery, King Abdulaziz University Faculty of Medicine, Jeddah, SAU

**Keywords:** spine, spinal cord, traumatic, gulf, sport

## Abstract

Traumatic spinal cord injuries (TSCIs) can lead to life-threatening consequences and neurological deficits. Sports activities significantly contribute to the incidence of these injuries. It is important to understand the epidemiology of sport-related spinal cord injuries (SCIs) in the Gulf region to improve patient care and increase awareness among this population. While studies from Saudi Arabia and Qatar have addressed SCIs related to both cycling and diving, we believe there is still significant scope for improvement in research on this topic. Special attention should be given to conducting retrospective studies across Gulf countries to establish a well-organized database.

## Introduction and background

Spinal cord injuries (SCI) substantially account for the overall trauma burden worldwide, despite comprising only 5% of all trauma cases [1]. There is a significant difference in the occurrence of SCI across various geographical regions. The incidence of SCI has been reported to range between 16 and 64 per 100,000 globally [1,2]. Epidemiological studies on SCI are constrained by small sample sizes and frequently rely on data from single institutions [2]. SCI is defined as the damage to the group of tightly packed cells and nerves that transmit signals between the brain and the rest of the body [1,2]. External causes of the injury are classified into the following six main categories: road traffic accidents (RTAs), falls, bicyclist-related Injuries, sports injuries, struck by or against an object, and others [firearms, machinery, pedestrians, transport (non-motor-vehicle collision-related), natural/environment-related] [2].

Acute spinal injuries are highly likely to occur during athletic activities. The risk of spine injury is elevated in several sports due to high speed, the potential for collisions, and traumatic falls. Athletes are frequently in close proximity to one another when competing and move at high speeds, further increasing the likelihood of injury [1,2]. Traumatic or acute spine injuries often occur during sports participation and are not rare. Lesions affecting the cervical spine pose the greatest risk of SCI and can lead to various neurological syndromes, representing a significant subset of sport-related injuries with the potential for long-term consequences as serious as paralysis or fatality [1,2]. The existing literature primarily examines SCI in the setting of the epidemiology of spine injuries, which is the most destructive type of spinal trauma. According to recent research, sports-related injuries constitute a substantial proportion of the 12,500 new SCIs that occur each year in the United States, accounting for about 9% of these incidents [2,3]. In addition, <1% of those injured fully recover at the time of their discharge from the hospital.

The extent of spinal cord damage depends on various factors, such as its location and the potential involvement of neural structures. A heightened level of suspicion, coupled with comprehensive history-taking, thorough physical examination, and appropriate imaging, is imperative to establish a proper diagnosis and formulate an effective management and treatment plan [2,3]. It is crucial to have both primary and secondary prevention strategies in place since there is no known cure for SCIs [1,3]. The objective of this analysis is to provide an overview of the current data on SCI epidemiology in sport-related incidents in the Gulf region.

## Review

Methods

Data Sources and Search Strategy

In conducting this systematic review, we adhered to the Preferred Reporting Items for Systematic Reviews and Meta-Analyses (PRISMA) guidelines. This study did not require an institutional board review approval since it incorporates information from previously published studies. A comprehensive search was performed to systematically explore literature across the MEDLINE Ovid, PubMed, EMBASE, and Web of Science databases, covering publications from the commencement of the study until June 17, 2021.

Study Selection

The initial screening of titles and abstracts yielded by the search was done by two independent investigators (L.A. and T.H.) on Endnote. Study selection was based on the following eligibility criteria: (a) traumatic spinal injury related to sports; (b) the study conducted in one of the Gulf region countries (Saudi Arabia, Kuwait, UAE, Qatar, Oman, and Yemen); and (c) studies in the English language. The exclusion criteria were as follows: systematic reviews, conference papers, and studies not in English. A manual web search was conducted to add any articles that did not appear in our primary search. 

Data Extraction and Statistical Analysis

The following information was extracted from the studies: author; title; year of publication; and demographic information, including age, gender, and country. Also, data related to the sport played, injury type, mechanism of injury, clinical presentation, radiological findings, treatment, early/late complications, outcomes, and follow-up were gathered. All available information was documented in a shared spreadsheet filled out by L.A. and T.H. The data were then summarized and statistically analyzed using Microsoft Excel version 16.72.

Results

**Figure 1:**
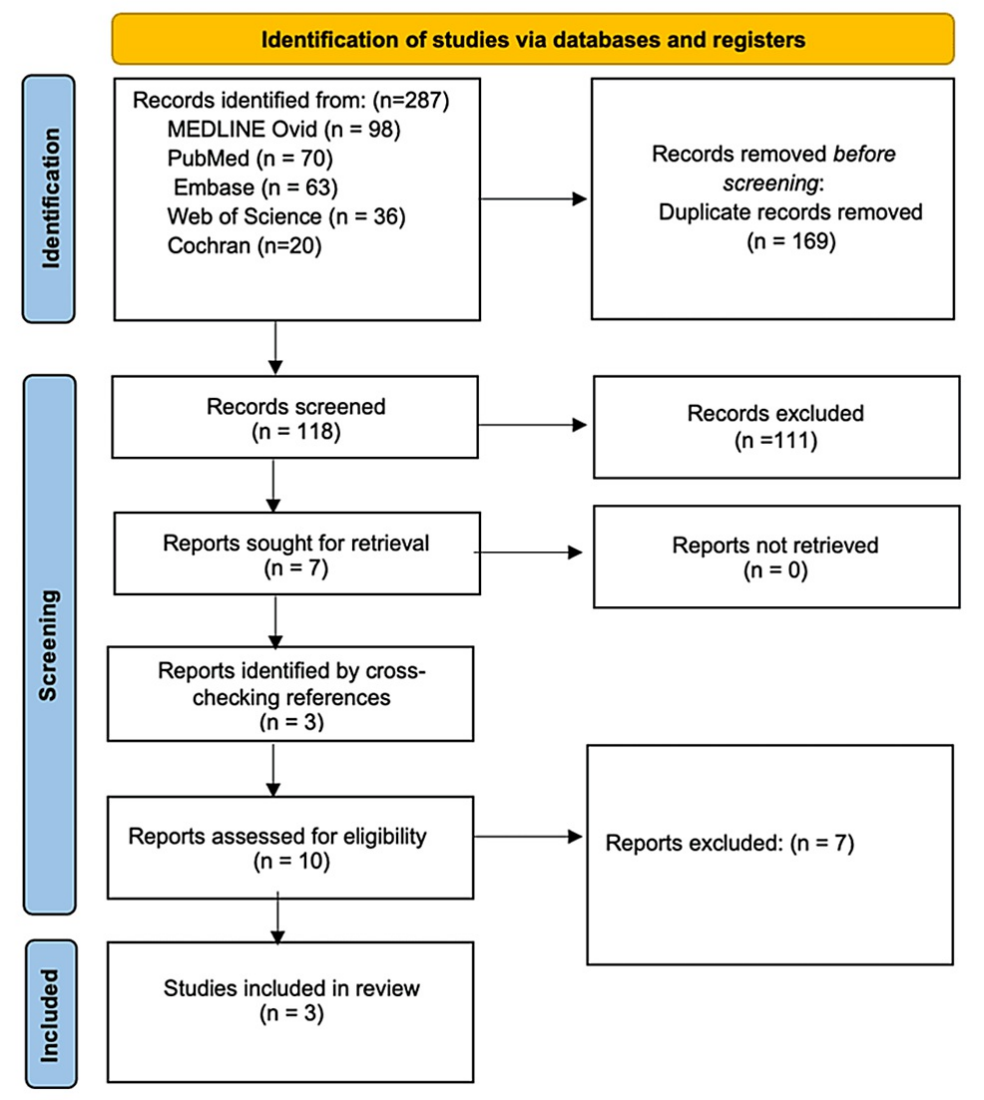
PRISMA flow diagram depicting study selection PRISMA: Preferred Reporting Items for Systematic Reviews and Meta-Analyses

Our search initially yielded 287 articles, distributed as follows: 98 from MEDLINE Ovid, 70 from PubMed, 63 from Embase, 36 from Web of Science, and 20 from Cochrane. Using EndNote Reference Manager (Version 20.5), we detected and eliminated duplicate articles, resulting in 118 unique articles. After screening titles and abstracts based on predetermined inclusion and exclusion criteria, we selected seven articles for full-text assessment and excluded 111 articles. Additionally, three articles were added from a manual web search, resulting in a total of 10 articles for full-text assessment. Following careful examination and adherence to the inclusion and exclusion criteria, three articles were deemed suitable for final inclusion, while seven were excluded due to a lack of mention of spinal injuries and failure to address sport-related injuries. This process is detailed in the PRISMA flow diagram in Figure 1. The findings suggest a dearth of data concerning sport-related traumatic SCIs (TSCIs) in Gulf countries. This investigation was undertaken to assess the available data and to ascertain the need for further research in this field.

Table 1 outlines a retrospective cohort study conducted in Qatar in 2019 by Abdelrahman et al. [4]. The study examines bicycle-related traumatic injury hospitalizations over six years. It encompasses 150 cases, primarily males, with a mean age of 27.2 years. The injuries resulted from collisions with cars or falls from bicycles. However, specific clinical and radiological findings, medical treatments, surgical interventions, and complications were not provided, warranting further investigation into outcomes and rehabilitation strategies.

**Table 1:** Summary of a study on bicycle-related traumatic injury hospitalizations

Author	Year	Study design	Country	Title	No. of patients	Age	Gender	Sport	Injury type	Mechanism of injury	Clinical presentation	Outcome
Abdelrahman et al. [4]	2019	Retrospective cohort study	Qatar	Bicycle-related traumatic injury hospitalizations: six years descriptive analysis in Qatar	150	Mean age: 27.2 ±16.6 years	147 males/3 females	Bicycle	N/A	Collision with a car or fall from a cycle	N/A	N/A

Table 2 summarizes the features of a study from Saudi Arabia. It was a retrospective cohort study conducted in 2021 by Alnaami et al., focusing on traumatic SCIs among motorsports participants [5]. The study involves 112 cases, primarily males, with an average age of 32.1 years. It delineates various American Spinal Injury Association (ASIA) classifications upon admission and presents the changes in ASIA classification post-treatment. However, details regarding radiological findings, medical and surgical interventions, and late complications were not specified, necessitating additional investigation into treatment modalities and long-term outcomes.

**Table 2:** Summary of a study on traumatic spinal cord injury from southern Saudi Arabia ASIA: the American Spinal Cord Injury Association

Author	Year	Study design	Country	Title	No. of patients	Age	Gender	Sport	Injury type	Clinical presentation	Outcome
Alnaami et al. [5]	2021	Retrospective cohort study	Saudi Arabia	Traumatic spinal cord injury in southern Saudi Arabia: Patterns, time to surgery and outcomes	112	Mean age: 32.1 ±14.12 years	101 males/11 females	Motorsport	ASIA type at admission: A: 34, B: 6, C: 16, D: 7, E: 49	Complete paralysis (34), weakness (22), numbness (9), back pain (65), impaired breathing (3)	39 patients had their ASIA classification improved by one class or more, 5 patients worsened, and 68 remained the same

Table 3 provides a summary of a retrospective study conducted in Saudi Arabia in 2012 by Alshahri et al., which examines TSCIs with a particular focus on diving-related incidents [6]. The study comprises 307 cases, predominantly male, with a mean age of 29.5 years. It highlights incomplete tetraplegia as a common outcome among diving-related TSCIs. However, detailed information on clinical presentations, radiological findings, medical and surgical interventions, and outcomes were not provided, indicating the need for further exploration of treatment approaches and complications associated with TSCIs.

**Table 3:** Summary of the study titled "Traumatic Spinal Cord Injury in Saudi Arabia: An Epidemiological Estimate From Riyadh"

Author	Year	Study design	Country	Title	No. of patients	Age	Gender	Sport	Injury type	Clinical presentation
Alshahri et al. [6]	2012	Retrospective study	Saudi Arabia	Traumatic spinal cord injury in Saudi Arabia: an epidemiological estimate from Riyadh	307	Mean age: 29.5 years	271 males/36 females	Diving	Traumatic spinal cord injury	Incomplete tetraplegia

Discussion

SCIs are a serious concern as they are associated with significant morbidity and mortality. Sport-related SCIs are the fourth most common cause of TSCIs worldwide, with motor vehicle collisions being the most common [7-13]. A significant incidence of sport-related SCIs has been observed in the United States, with 9% of all new SCIs being connected to sports. Spine injuries and cervical spine fractures account for many SCIs, which lead to permanent severe functional disability or severe head or neck trauma with no permanent consequences; these injuries are very common in sports [13]. Athletes in different types of sports experience a high incidence of SCIs.

In 2014, The Stockholm Spinal Cord Injury Study put in place a surveillance system to record newly admitted patients with TSCIs. This led to an improvement in the organization of healthcare services for newly injured patients and those diagnosed with TSCI [7-11]. To better understand the frequency and distribution of TSCIs in different sports around the Gulf region, doctors need to establish a local database containing all reported cases. However, several questions remain regarding its feasibility or affordability [7]. The availability of epidemiological figures can be beneficial in providing healthcare professionals with relevant information, which can aid in better treatment planning, understanding the types of injuries and associated outcomes, and improving preventive strategies and athlete awareness [7,8].

It is imperative to take action to reduce TSCIs in sports. To do so, strict enforcement of game rules, teaching fundamental skills, and educating athletes, parents, and coaches about the signs and symptoms of head injuries are vital [8]. Participation in pre-event medical exams and hiring certified athletic trainers must be mandatory at every level of competition. Athletes showing signs of head trauma must receive immediate medical attention and should only return to participation after being cleared by a physician or certified athletic trainer [9-11]. An emergency plan must be in place and the details distributed to all relevant personnel, outlining evacuation plans, available transportation, open and portable communication, and game and practice schedule awareness in the local hospital emergency departments. These measures may not prevent injuries entirely, but they can potentially prevent severe injuries causing permanent disability [8,9].

Records released by the Ministry of Health indicate that Saudi Arabia has one of the highest rates of SCIs in the world, mainly due to RTA, while there are no statistics on sport-related injuries [8]. Further research is needed to focus on the prevalence of sport-related TSCIs in the region. Gathering data on the prevalence of these injuries could aid in improving the quality of life and overall function, as seen in Sweden, where preventive measures against TSCI have been prioritized based on the collected data [8-9]. Currently, most of the available data on sport-related TSCIs were collected from Qatar and Saudi Arabia, specifically in the sports of cycling and diving. However, the level of awareness on the topic of TSCI in the Gulf is on the rise. 

This study has a few limitations, and future studies should try to address them The included studies only involved individuals aged 18 and older, highlighting the need for more data on pediatric TSCI cases. As a result, the number of cases in this study may not accurately reflect the extent of TSCI morbidity in this particular region of the Gulf. Moreover, the incidence rate reported in this study could be either an overestimation or an underestimation [11].

There is still a lack of data regarding TSCI among athletes in the Gulf, coupled with a lack of resources to establish the epidemiology in the region, which could be rectified by conducting a large retrospective study across several countries in the Gulf, to establish an extensive database that offers comparative data of cases among different countries. Finally, such a study would benefit hospitals, clinicians, and the ministries of health in providing optimal care to patients suffering from TSCIs [11].

## Conclusions

TSCIs are a major health concern, often leading to severe disability and life-altering consequences, with sports-related activities being a prominent risk factor, often resulting in complete paralysis. Our study focused on the epidemiology of TSCI among athletes in the Gulf countries. This systematic review aimed to uncover the prevalence, characteristics, and outcomes of sport-related SCIs in the Gulf region, revealing valuable insights, particularly in countries like Saudi Arabia and Qatar. However, significant gaps in data availability, particularly the absence of studies from other Gulf nations, were noted. Despite the importance of comprehending the epidemiology of TSCI in sports, our analysis identified limitations in the available evidence, including a scarcity of comprehensive studies and standardized reporting frameworks. Hence, our study highlights the urgent need for further research to improve our understanding of sport-related SCIs in the Gulf region. Future studies should prioritize comprehensive data collection, standardized reporting practices, and multi-country collaborations to generate robust evidence and inform evidence-based interventions.
